# A retrospective study of thermal events on the mortality rate of hutch-reared dairy calves

**DOI:** 10.3389/fvets.2024.1366254

**Published:** 2024-03-15

**Authors:** Viktor Jurkovich, Mikolt Bakony, Jeno Reiczigel

**Affiliations:** ^1^Centre for Animal Welfare, University of Veterinary Medicine, Budapest, Hungary; ^2^Department of Animal Hygiene, Herd Health and Mobile Clinic, University of Veterinary Medicine, Budapest, Hungary; ^3^Centre for Translational Medicine, Semmelweis University, Budapest, Hungary; ^4^Department of Biostatistics, University of Veterinary Medicine, Budapest, Hungary

**Keywords:** dairy calves, heat stress, mortality, heatwaves, calf days, risk ratio

## Abstract

**Introduction:**

Heat stress in hutch-reared dairy calves (*Bos taurus*) is highly relevant due to its adverse effects on animal welfare, health, growth, and economic outcomes. This study aimed to provide arguments for protecting calves against heat stress. It was hypothesized that the thermal stress caused by high ambient temperature in summer months negatively affects the survival rate in preweaning calves.

**Methods:**

In a retrospective study, we investigated how calf mortality varied by calendar month and between thermoneutral and heat stress periods on a large-scale Hungarian dairy farm (data of 46,899 calves between 1991 and 2015).

**Results:**

The daily mortality rate was higher in the summer (8.7–11.9 deaths per 10,000 calf days) and winter months (10.7–12.5 deaths per 10,000 calf-days) than in the spring (6.8–9.2 deaths per 10,000 calf-days) and autumn months (7.1–9.5 deaths per 10,000 calf-days). The distribution of calf deaths per calendar month differed between the 0–14-day and 15–60-day age groups. The mortality risk ratio was highest in July (6.92). The mortality risk in the 0–14-day age group was twice as high in periods with a daily mean temperature above 22°C than in periods with a daily mean of 5–18°C.

**Conclusions:**

Heat stress abatement is advised in outdoor calf rearing when the mean daily temperature reaches 22°C, which, due to global warming, will be a common characteristic of summer weather in a continental region.

## 1 Introduction

On large-scale dairy farms in Hungary, as in many dairy cattle (*Bos taurus*) farms worldwide (1, 2), preweaning calves are housed outdoors in hutches. In summer, it is usual that calves are inactive, cuddled up in the shaded area of the hutch and panting at a very high rate (3, 4). Beyond the apparent welfare concern, heat stress can negatively affect animal health and productivity. Describing the adverse effects of heat stress experienced by hutch-reared calves and the advantages of heat stress abatement have been the topic of an increasing number of studies recently [see reviews of Roland et al. (1) and Bakony and Jurkovich (5)].

Stress, including heat stress, has a biological cost; that is, the energy requirement of adaptive mechanisms being shifted away from growth or production (6). Indeed, the average daily preweaning weight gain of hutch-reared calves was shown to be lower in seasons with high average temperatures (7). A decrease in starter intake caused by prolonged inactivity and discomfort can also contribute to a slower rate of growth (8, 9).

Heat stress in the preweaning period can also compromise the immune response (10, 11). High temperatures may be particularly challenging for newborn calves due to immature thermoregulation and low innate immunity (11, 12). The overall effect of such adverse thermal effects can lead to increased mortality of hutch-housed calves (13, 14), affecting different age groups differently (15). However, contradicting findings on the mortality rate have also been published (16, 17), which led us to investigate the phenomenon further.

In a retrospective study, we investigated how mortality rates of preweaning calves were influenced by season on a commercial dairy farm. We tried to quantify the differences in mortality rates between periods of presumed heat stress and thermoneutrality. By collecting data from a single farm, we aimed to keep the management factors that could influence calf mortality (18, 19) as constant as possible.

## 2 Materials and methods

### 2.1 Data acquisition

Data were retrieved from the farm management database of Enyingi Agricultural Ltd. (Kiscséripuszta, Hungary, 47°02′12.5“N 18°21′30.1”E), with the agreement of the farm management, referring to the period from January 1991 to December 2015. The farm had an average animal population of 1,500–1,800 Holstein Friesian cows and their offspring in the studied period. The farm purchased no calves or pregnant heifers; only the offspring of their cows were reared. The database contained information on each calf born on the farm, regarding—among others—date of birth, estimated birth weight, sex, and date of leaving the calf population (due to sales, death, transportation to the heifer group, etc.). When considering any exclusions, the factors that could act as confounders were considered. Sex was found not to be confounding with mortality because the rearing protocol (see later) did not differ between heifer and bull calves, and any effect of sex on perinatal mortality is not fully elucidated (20). Regarding birth weight, no inclusion threshold was set. Firstly, birth weights were generally based on the educated guess of the experienced stockperson and always rounded to the nearest multiple of 5 kg (25 kg, 30 kg, etc.). Second, the effect of heat stress (which the study aimed to investigate) could manifest in lower birth weights, which can be associated with lower viability (21–23). Stillbirths (death occurring within 24 h after birth) were also not excluded so as not to miss out on fatal heat shocks occurring in extremely hot weather that can kill outdoor-kept newborn calves (24), especially those born to cows with dystocia, that occur more frequently in heat-stressed dams (21). Twins were also not excluded due to the assumption that birth weight already represents the effect of twin pregnancy on calf mortality, and the clinical relevance of the season-related differences in twin pregnancies is questionable (25).

Finally, a database containing data on 46,899 calves was obtained, of which 2,155 calf deaths were recorded at the farm, occurring before the age of 60 days. A dataset was created that contained for every single day between 1 January 1991 and 31 December 2015 the number of 0–60 day-old calves alive and the number of 0–60 day-old calves deceased.

### 2.2 Calf housing and calf rearing protocol

Within 4 h of birth, calves were given 10% of their body weight in good quality colostrum, mainly from the mother; in case the colostrum did not have the required IgG content (Brix >23), they received frozen colostrum of controlled quality. Afterwards, the calves were transferred to the individual hutches outdoors (except for exceptionally cold winters, when newborns spent 2 days in the calving barn in individual hutches before transfer). Outdoor hutches were made of wood with slate roofs and were bedded with straw. From day 1, milk replacer was fed from buckets twice daily, 4 liters per meal. Calf starter and water were provided *ad libitum* from day 4. IgG supply was checked between 2 and 5 days of age with a Brix-refractometer. The calves received no vaccination; instead, the dams were vaccinated in the dry period against *Escherichia coli*, adenovirus, rotavirus and coronavirus. Weaning took place around 60 days of age by gradually diluting the milk replacer for a few days until complete weaning.

The farm did not apply seasonal heat or cold abatement strategies in calf rearing. The location and the type (material) of calf hutches did not change throughout the study period, even during necessary repurchases. The same veterinarian was employed on the farm for the first 17 years of the observation period. The changes in calf-rearing technology and protocol concerned the source (manufacturer) of milk replacer (with similar composition). Since all management changes affected all calves similarly all year round, these were found not to be confounding with the potential effects of the season (reflected by specific temperature conditions or calendar months) on mortality.

### 2.3 Thermal data and events

Meteorology data were collected from the National Centers for Environmental Information (Asheville, NC, USA; https://www1.ncdc.noaa.gov/pub/data/gsod/), using the data from the Hungarian Meteorological Service station nearest to the farm (Siófok, Hungary, 46°54′35.1“N 18°02′41.2”E). The meteorological station was 25 km from the farm in a plain region. Weather data included daily mean, minimum and maximum temperatures calculated from hourly dry bulb temperature measurements for each day between 1 January 1999 and 31 December 2015.

**Figure 1:**
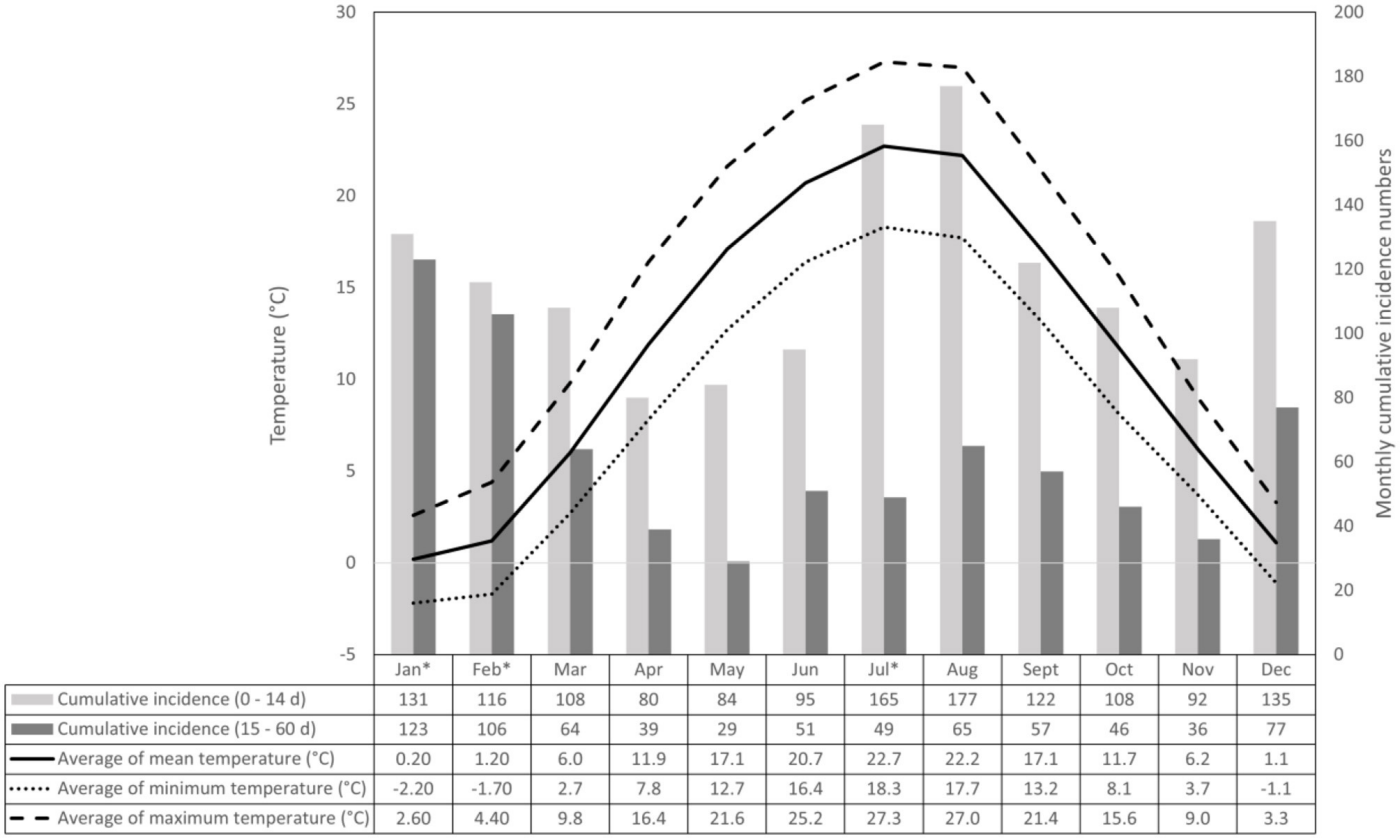
Monthly cumulative incidence number of calf deaths in the two age groups and monthly averages of mean, minimum and maximum ambient temperatures. Asterisks indicate months that contributed significantly to the difference in the annual distribution of monthly cumulative incidence number between the age groups (*p* < 0.01).

Dry bulb temperatures were considered adequate to determine heat stress in dairy calves. Relative humidity does not add much to the informative value of ambient temperature in assessing the thermal load of dairy calves reared outdoors in a continental region (26, 27). Periods of heat stress, heat waves and thermoneutrality were distinguished to define risk and reference periods for risk assessment. Days with a mean daily temperature not lower than 22°C were considered heat stress days (27). According to the Hungarian Meteorology Institution, heat wave days occur in Hungary when the daily mean temperature exceeds 25°C, and heat waves occur when there are at least three heat wave days. Therefore, days with daily mean temperatures not lower than 25°C were considered heat wave days. Different ambient temperatures were reported as set points of increased evaporative heat dissipation in dairy calves. Gebremedhin et al. (28) observed increased respiration as temperature exceeded 20°C, while other researchers agreed on 26°C as the upper critical temperature of preweaned calves (29, 30). We chose the thermoneutral reference periods (mean daily temperature between 5 and 18°C) to be lower than the lowest upper critical temperature in the literature (29) and higher than the highest lower critical temperature (31). Also, these temperature thresholds correspond well with the mean daily temperatures (Figure 1) of spring (March–May) and autumn (September–November) months in Hungary, which has a continental climate.

### 2.4 Data analysis

The temperature data were paired to each day in the calf dataset (the calendar days and the referring number of living and deceased calves 0–60 days of age). Two other datasets were created based upon the first; one contained the same data, but the age was restricted to 0–14 days, and in the other, the age limits were 15–60 days.

First, we calculated average daily mortality rates in each calendar month for the whole preweaning period (0–60 days) and separately for the 0–14 days age group and the 15–60 days age group. The number of births and deaths was available each day from 1 Jan 1991 to 31 December 2015. Since the calf population was an open population, the number of animals varied daily due to births, deaths, weaning or sales. Thus, we defined the number of calves at risk for a given period via calf-days (32). According to this, the average daily mortality rate in a given period was calculated by dividing the number of deaths by the sum of calf-days in that period. We calculated the daily mortality rates per 10,0000 calf-days. It can be taken in case of e.g., 6.8 as an average mortality rate of 0.068 % daily. Over the first 60 days of life, for example (e.g. the approximate length of the preweaning period), the risk is 0.068% ^*^ 60 = around 4.1%. By adding up the daily mortality rates, we can estimate the average mortality risk in any given (60-day) period in the year. The risk of mortality of a particular calf depends on which period of the year its preweaning period falls into. The daily mortality rates calculated for the calendar months were compared between the age groups [0–14 days vs. 15–60 days by calculating risk ratios, with 15–60 days as the reference category (Table 1)].

**Table 1:** Average daily mortality rate of calves (per ten thousand) around the year in total and by age groups.

**Age group**	**Jan**	**Feb**	**Mar**	**Apr**	**May**	**June**	**July**	**Aug**	**Sep**	**Oct**	**Nov**	**Dec**
0–60 days	12.4	12.5	9.4	7.2	6.8	8.7	11.1	11.9	9.5	8.4	7.1	10.7
0–14 days	20.7	22.1	20.3	16.2	15.9	17.5	26.3	28.5	22.3	19.6	16.2	21.8
15–60 days	8.7	8.5	5	3.4	2.5	4.5	3.8	4.6	4.2	3.6	2.9	5.7
^a^RR_inc.rate_	2.37	2.60	4.06	4.76	6.36	3.88	6.92	6.19	5.31	5.44	5.58	3.82

^a^Risk ratio (RRinc.rate) is the ratio of the incidence rate of the 1st age group divided by that of the 2nd one.

Then, the monthly cumulative incidence of deaths was summarized for each calendar month. Monthly cumulative distributions mean how all calf deaths occurring throughout the 25 years are distributed between calendar months and age groups, that is, how many of the 2,155 deaths occurred in the month of January, February, etc., separately for the 0–14 day-old and 15–60 day-old calves. We applied the chi-squared test to compare the monthly distribution of cumulative incidence numbers in the two age groups. The adjusted standardized residuals were computed to explore which months contributed most to the difference (33). The *p*-values were corrected for multiplicity by the Bonferroni-Holm method (34).

Second, we compared the average mortality rates of the first age group (0–14 days) in heat stress and thermoneutral periods by Fisher's exact test. For comparison, we repeated the analysis with 4-day and 5-day periods and temperature thresholds of 23, 24, 25 and 26°C (heat wave periods).

All statistical computations were carried out by R 3.5.2 (35).

## 3 Results

In the studied 25-year period, the average mortality rate of calves younger than 2 months was 9.64 per ten thousand calf-days, exhibiting elevated mortality rates in the winter and summer months (Table 1). The mortality risk ratio of the age group 0 to 14 days compared to the rest (15–60 days) was above 2 throughout the year (Table 1). It was highest in July (6.92), the hottest month in Hungary, and lowest in January (2.37). Monthly average, maximum and minimum temperatures and cumulative incidence of calf deaths in 0–14 day and 15–60 day age groups are summed up in Figure 1. The chi-square homogeneity test found a significant difference between the distribution of monthly cumulative incidence numbers in the two age groups (X^2^ = 67.362, df = 11; *p* < 0.0001). The Bonferroni-Holm-corrected adjusted standardized residuals detected the difference as significant in 3 months, namely in the coldest (January, February; p < 0.001, respectively) and hottest (July, *p* = 0.0018) month of the year (Figure 1). In accordance with the mortality rates in Table 1, cumulative incidence proved to be highest in July in the age group of 0 to 14 days. In contrast, it was highest in the winter months among older calves.

The average mortality risk and odds ratios in the 0–14 day age group are displayed in Table 2, along with the defining parameters. The mortality risk in the heat stress periods was at least twice as high as in the thermoneutral reference periods. With a daily mean temperature of 25°C or more (heat waves), the risks were three times as high as in the reference period. Varying the reference length and risk periods did not substantially change the calculated measures of association.

**Table 2:** Reference and heat stress periods and risk ratios.

**Length (days)**	**No. of reference periods^a^ (no. of days in ref. periods)**	**Mortality risk in the reference periods (per 1,000 calf-days)**	**Minimum daily mean temperature in the risk periods (°C)**	**No. of risk periods (No. of days in risk periods)**	**Risk ratio**	**Odds ratio**	**Odds ratio confidence interval**	***p*-value**
3	943 (2,829)	1.76	22	248 (744)	2.07	2.07	1.74; 2.46	<0.0001
			23	165 (495)	2.32	2.33	1.92; 2.81	
			24	109 (327)	2.53	2.54	2.04; 3.15	
			25	63 (189)	3.00	3.01	2.32; 3.86	
			26	32 (96)	3.54	3.56	2.56; 4.84	
4	625 (2,500)	1.72	22	152 (608)	2.05	2.05	1.69; 2.48	
			23	102 (408)	2.28	2.28	1.84; 2.81	
			24	64 (256)	2.65	2.66	2.08; 3.37	
			25	34 (136)	3.39	3.40	2.54; 4.49	
			26	19 (78)	3.15	3.16	2.12; 4.57	
5	461 (2,305)	1.80	22	102 (510)	1.94	1.94	1.58; 2.38	
			23	64 (320)	2.20	2.21	1.75; 2.77	
			24	32 (160)	2.58	2.59	1.92; 3.44	
			25	15 (75)	3.50	3.52	2.43; 4.97	
			26	7 (35)	3.37	3.38	1.94; 5.52	

^a^Minimum and maximum daily mean temperature in the reference periods were between 5°C and 18°C.

## 4 Discussion

The temperature conditions measured in the studied farm were outside the thermoneutral zone of dairy calves in the summer and winter months. Considering that the geographic location was nothing extreme in terms of, e.g., topography or altitude above sea level, the annual changes in the average ambient temperatures can be regarded as representative of the country and, in broader terms, regions having a dry continental climate. In calves, exposure to suboptimal temperatures may induce thermal stress and related health events (1, 36). In our study, the average daily mortality rates of preweaning calves were shown to deviate from the overall average in those calendar months where the mean temperatures differed from the optimal, suggesting an association between environmental conditions and the viability of hutch-reared calves. Beyond environmental stressors, calf mortality in the postnatal period is influenced by numerous factors, including *in-utero* stress, calving difficulty, newborn calf management, nutrition, housing quality, etc. (15, 21). It is an apparent limitation of our study that most of these factors were not recorded individually for each newborn calf; however, all reasonable efforts for standardization were made by choosing a single farm where no pregnant heifers or calves were ever purchased, and management factors were known to be relatively constant; and by collecting an impressive amount of data to even out periods of potential disease outbreaks or extreme weather events. Comparing the annual distributions could have highlighted potential peaks in mortality in a particular month or year that could have been traced back to some unfavorable event or condition. However, the incidence numbers of monthly death cases would have been very low when broken down to each year of the study period (see the magnitude of 25-year cumulative incidence numbers in Figure 1). Such low numbers are favorable from an animal health point of view but cannot provide sufficient power for statistical comparisons.

One could also argue that wooden hutches are outdated compared to modern-day calf hutches, and it could indeed impact mortality rates. Although disinfection of wooden hutches is more cumbersome than that of plastic hutches, their thermal properties are very favorable, and no disadvantages concerning the general health status of indwelling calves were shown in comparison with polyethylene hutches (37, 38). In light of the above, the hutch material could affect overall calf welfare, but not the seasonal variations in mortality, since the material did not change during the studied period.

Concerning the second analysis, the annual number of risk and reference periods were even throughout the 25-year study period (data are not displayed). Therefore, we could rule out that the exceptionally hot summers in a few of the years would be responsible for increased calf mortality. We did not set the aim of defining a specific threshold for heat stress. Instead, we aimed to justify the need for heat stress abatement by confirming a positive association between mortality rate and high temperatures accepted as indicators of heat stress or heat waves. Compared to thermoneutrality, a mean daily temperature of 22°C or above for 3–5 consecutive days (heat stress) was associated with a 97–107 % increase in calf mortality (Table 2). Mean daily temperatures above 25°C for at least three consecutive days (heat waves) were associated with a mortality risk three times higher than thermoneutral reference periods (Table 2). We thus concluded that the weather conditions could have affected the observed temperature-related changes in preweaning calf mortality rates. The highest risk ratios of calf deaths in the 0–14 days age group vs. the 15–60 day old age group were observed in the summer months, while the lowest risk ratios were observed in winter. It may indicate that newborn calves are more susceptible to high environmental temperatures, presumably due to their undeveloped thermoregulation. At the same time, cold stress reduces survival in both age groups, supposedly due to respiratory diseases. Although using the temperature humidity index (THI) is widespread to assess the thermal environment, we decided not to use it for two main reasons. First, the THI is the weighted average of DBT and relative humidity. It was originally developed for humans and later used in animal studies, mostly in cases of lactating dairy cows in barn environments. Currently, there are several different equations for calculating the THI (39). We have no accurate knowledge of how relative humidity affects the thermal perception of pre-weaning calves; therefore, we have no clear idea of an appropriate weighting factor. Second, it has been shown earlier that DBT has a stronger correlation with the heat stress response of dairy calves than most of the THIs commonly used to assess thermal stress in pre-weaning calves (27, 40).

Our findings are in accordance with Martin et al. (13), who showed that ambient temperatures are among the environmental factors that increase the mortality rate of calves. In a more recent study (14), the data of rendering companies showed that the relationship between mortality in the preweaning period and average temperature follows a U-shaped curve. Higher calf mortality rates were associated with an average daily temperature above 24°C (14). The arrival of calves to a veal facility in summer was also a risk factor for increased mortality (41). The 2003 and 2006 heatwaves in France were associated with increased mortality rates of 0–7-day-old and 8–60-day-old dairy calves (42).

However, it has also been reported that it is the first and last quarter of the year that takes a higher toll on calf health across US dairies (16), which contradicts our results. We found our study design not directly comparable to that of Wells et al. (16). They investigated the average death incidence rate in the quarters of the year, not the seasons. Months within each quarter can have a substantially different impact on calf mortality. Furthermore, a possible interaction between the season of calf birth and the dairies' geographic region was not investigated, which could have influenced the results. Urie et al. (17) also found that the preweaning calf mortality rate negatively correlates with the average temperature-humidity index. However, their study involved only 1 year, in which weather events or other conditions could differ from the usual and could thus confound the results.

High temperatures at birth usually go together with high temperatures in the last gestation phase. Consequently, the direct effects of hot weather on the newborn calf may be coupled with the carryover effect of maternal heat stress. Heat stress *in utero* can lead to intrauterine growth retardation, adversely affecting the newborn's adaptive skills (21, 23). The shortening of the gestation length (41, 43) also affects calf viability. Heat stress makes dry cows potentially more prone to dystocia, which increases the risk of stillbirth and death before 120 days of age (44, 45).

The main limitation of our study is that we collected the data from only one farm. This way, we tried to keep the influential management factors as constant as possible to avoid farm effects. The large dataset may counterbalance this limitation. Another limitation is that the meteorological data were measured at a meteorological station 25 km distance from the farm, not on the spot, and not even in the calf hutches. Based on our previous studies (3, 4), we know that the dry bulb temperature is higher in the hutch than outside. Since this was a retrospective study, we could not measure the micro-climate in the inner hutch environment. Another study (46) also used the same set-up (retrospectively comparing the dairy milk yield of this farm with the data of the same meteorological station we used) and successfully drew important conclusions.

## 5 Conclusions

Heat stress was shown to be associated with calf mortality during summer, especially among the young (0–14-day-old) calves. The mortality risk increased twofold when the mean daily temperature exceeded 22°C and threefold when the heat waves (daily mean temperature higher than 25°C) occurred. Heat stress abatement is advised in outdoor calf rearing when the mean daily temperature reaches 22°C, which, due to global warming, will be a common characteristic of summer weather in a continental region.

## Data availability statement

The raw data supporting the conclusions of this article will be made available by the authors, without undue reservation.

## Ethics statement

Ethical review and approval was not required for the study on animals in accordance with the local legislation and institutional requirements. Written informed consent was obtained from the owners for the participation of their animals in this study.

## Author contributions

MB: Conceptualization, Data curation, Formal analysis, Investigation, Methodology, Writing – original draft, Writing – review & editing. VJ: Conceptualization, Funding acquisition, Investigation, Supervision, Writing – original draft, Writing – review & editing. JR: Formal analysis, Investigation, Methodology, Writing – original draft.
